# GRAND: An Integrated Genome, Transcriptome Resources, and Gene Network Database for *Gossypium*

**DOI:** 10.3389/fpls.2022.773107

**Published:** 2022-01-21

**Authors:** Zhibin Zhang, Mao Chai, Zhaoen Yang, Zuoren Yang, Liqiang Fan

**Affiliations:** ^1^State Key Laboratory of Cotton Biology, Institute of Cotton Research, Chinese Academy of Agricultural Sciences, Anyang, China; ^2^Zhengzhou Research Base, State Key Laboratory of Cotton Biology, Zhengzhou University, Zhengzhou, China

**Keywords:** *Gossypium*, genome, comparative genomics, variation, GRAND

## Abstract

With the increasing amount of cotton omics data, breeding scientists are confronted with the question of how to use massive cotton data to mine effective breeding information. Here, we construct a *Gossypium* Resource And Network Database (GRAND), which integrates 18 cotton genome sequences, genome annotations, two cotton genome variations information, and also four transcriptomes for *Gossypium* species. GRAND allows to explore and mine this data with the help of a toolbox that comprises a flexible search system, BLAST and BLAT suite, orthologous gene ID, networks of co-expressed genes, primer design, Gbrowse and Jbrowse, and drawing instruments. GRAND provides important information regarding *Gossypium* resources and hopefully can accelerate the progress of cultivating cotton varieties.

## Introduction

Cotton (*Gossypium* spp.) produces natural fiber for the textile industry worldwide and also plays an important role in edible oil for daily life. The *Gossypium* genus includes more than 50 different species, and it is an excellent model for studying genome evolution and polyploidization. Moreover, multiple high-quality *de novo* assembled genomes of *Gossypium* have been reported in recent years. These genomes have considerable improvements and contiguity compared to previously assembled draft genomes. For example, the high-quality genomes of *Gossypium arboreum* (Du et al., 2018), *Gossypium austral* (Cai et al., 2019), *Gossypium raimondii* and *Gossypium turneri* (Udall et al., 2019), *Gossypium davidsonii* and *Gossypium thurberi* (Yang et al., 2021) were sequenced and released in 2018, 2019, 2019, and 2021, respectively. Genomes of tetraploid *Gossypium barbadense* and *Gossypium hirsutum* were *de novo* sequenced and released by Hu et al. (2019) and Wang et al. (2019), respectively. Yang et al. (2019) also sequenced and assembled genomes of two upland cotton cultivars TM-1 and zhongmiansuo24 (ZM24). The assembly of these cotton genomes (diploid and tetraploid) transitioned *Gossypium* research into the genomics and pan-genomics era. However, effective integration and utilization of a large number of cotton datasets to mine valuable information for cotton researchers have become an important research hotspot.

Several online databases about cotton have been designed worldwide. CottonGen (Yu et al., 2014) is a relatively comprehensive cotton database with a collection of cotton genomes, genetic markers, and breeding germplasm accessions, while it is sometimes unfriendly for users and the functional modules need to be further expanded. ccNet (You et al., 2017) is a co-expression network database of diploid *G. arboreum* and polyploid *G. hirsutum*. CottonFGD (Zhu et al., 2017) is a cotton functional genome database, which integrates cotton genomes and transcriptomes as well as sequence retrieval, analysis, and visualization modules, but it does not contain genetic data, such as molecular markers. MaGenDB (Wang et al., 2020) focuses on constructing an integrative database of 13 Malvaceae species, including cotton, to enable users to jointly compare and analyze relevant data. COTTONOMICS^1^ is a comparative genomics platform and variation database for *G. hirsutum* and *G. barbadense*. CottonGVD (Peng et al., 2021) is a cotton database specifically focused on trait-associated loci visualization. Therefore, it is necessary to build a cotton database that systematically gathers the latest cotton genomes, transcriptomes, and molecular markers data together.

To meet this goal, here, we developed a comprehensive cotton database GRAND by integrating high-quality genomic and transcriptomic resources of cotton and providing tools for multi-level integrative analysis. GRAND covers a systematic view of genomic and transcriptomic information, integrates gene searching, gene list analysis, and visualization tools (such as Expression Visualization, Heatmap Draw, KEGG Dot Plot, and Annotation function). Besides, GRAND is an omics database for cotton (*Gossypium* spp.), in which all data can be freely accessed and downloaded.

## Materials and Methods

### Data Sources

The sequences of 18 cotton genome assemblies representing 14 *Gossypium* species and their respective gene annotations data, together with four transcriptomes were downloaded directly from relevant databases or sequenced by our laboratory and further used in GRAND (Table 1). In Table 1, the suffix of each species name indicates the institution that sequenced and published the genome [such as ICR: Institute of Cotton Research of CAAS, ZJU: Zhejiang University, HAU: Huazhong Agricultural University, USDA-ARS: US Department of Agriculture (USDA) Agricultural Research Service (ARS)]. The transcriptome data of *G. hirsutum_TM-1* and *G. barbadense_H7124_ZJU* were downloaded from the NCBI Sequence Read Archive with the accession number PRJNA490626 and were published by Hu et al. (2019) from Zhejiang University. The other two transcriptomes, *G. arboreum_ICR* and *G. hirsutum_ZM24_ICR*, were sequenced and assembled by Du et al. (2018) and Yang et al. (2019) of the Institute of Cotton Research (ICR), respectively. Illumina reads were aligned to references *G. hirsutum_TM-1_ICR* (Yang et al., 2019), *G. hirsutum_ZM24_ICR* (Yang et al., 2019), and *G. arboreum_ICR* (Du et al., 2018), respectively, using TopHat 2.1.1 (Kim et al., 2013). Quantification of gene expression was then performed with Cufflinks version 2.2.1.^2^ These data are shared freely on these websites without analysis tools to analyze them online, and the inconsistent format of the different datasets makes it more difficult to use them jointly. We have solved these problems and all the data are available for free download from GRAND.

**Table 1:** Summary of 18 cotton genome assemblies representing 14 *Gossypium* species.

Species	Genome size (Mb)	Number of protein-coding genes	Contig N50 (Mb)	Scaffold N50 (Mb)	References	Data sources
*G. barbadense*_ZJU	2,225	75,071	0.08	23.44	Hu et al., 2019	https://www.ncbi.nlm.nih.gov/bioproject/PRJNA450479/
*G. hirsutum*_TM-1_ZJU	2,295	72,761	0.11	15.51		
*G. raimondii*_USDA-ARS	735	40,743	6.3	6.3	Udall et al., 2019	https://www.ncbi.nlm.nih.gov/bioproject/PRJNA493304
*G. barbadense*_HAU	2,266	71,297	2.15	92.88	Wang et al., 2019	https://www.ncbi.nlm.nih.gov/bioproject/?term=PRJNA433615
*G. hirsutum*_TM-1_HAU	2,347	70,199	1.89	97.74		
*G. arboreum*_ICR	1,710	40,960	1.10	NA	Du et al., 2018	https://www.ncbi.nlm.nih.gov/bioproject/PRJNA382310
*G. hirsutum*_TM-1_ICR	2,286	73,624	4.76	NA	Yang et al., 2019	https://www.ncbi.nlm.nih.gov/bioproject/PRJNA503326/
*G. hirsutum*_ZM24_ICR	2,309	73,707	1.98	NA		
*G. australe*_ICR	1,752	40,694	1.83	143.6	Cai et al., 2019	https://www.ncbi.nlm.nih.gov/bioproject/?term=PRJNA513946
*G. davidsonii*_ICR	801	41,471	26.8	NA	Yang et al., 2021	http://grand.cricaas.com.cn/page/download/download
*G. thurberi*_ICR	780	41,316	24.7	NA		
*G. tomentosum*	2,229	72,648	11.98	103.05	Shen et al., 2021	https://www.ncbi.nlm.nih.gov/bioproject/PRJNA629964
*G. darwinii_v1.1*	2,210	78,303	9.07	101.9	Chen et al., 2020	https://www.ncbi.nlm.nih.gov/bioproject/PRJNA516409/
*G. mustelinum*	2,344	74,699	2.31	106.76		https://www.ncbi.nlm.nih.gov/bioproject/?term=PRJNA525892
*G. anomalum_NSF_B1*	1,208	37,016	10.81	97.68	Grover et al., 2021	https://www.ncbi.nlm.nih.gov/bioproject/PRJNA421337
*G. herbaceum__WHU*	1,572	43,952	1.91	117.88	Huang et al., 2020	https://www.ncbi.nlm.nih.gov/bioproject/?term=PRJNA506494
*G. longicalyx*	1,205	38,378	17.53	95.88	Grover et al., 2020	https://www.ncbi.nlm.nih.gov/bioproject/PRJNA420071
*G. turneri-TURN-v1.0*	765	39,692	7.91	60.46	Udall et al., 2019	https://www.ncbi.nlm.nih.gov/bioproject/PRJNA493521

### Development of Database and Website

The GRAND database relies on the Linux operating system, using J2EE as the framework, MySQL as the back-end database, and Apache Tomcat as the server. Genome sequence, annotation, expression, and variation data are stored in the MySQL database. A web interface based on JavaServer Pages (JSP), HTML5 and, CSS3 is constructed to enable end-users to access GRAND data through any modern browser on any kind of device. The GRAND database is hosted on a server equipped with eight 14-cores Intel Xeon Gold 5120 processors.

### Orthologous Gene ID Function and Gene Network

The orthologous genes among *G. hirsutum* TM-1 and ZM24 (Yang et al., 2019), *G. hirsutum* TM-1 (Hu et al., 2019), *G. barbadense* (Hu et al., 2019), *G. arboreum* (Du et al., 2018), *G. hirsutum* TM-1 (Wang et al., 2019), and *G. barbadense* (Wang et al., 2019) were identified using Inparanoid v4.1 (O’Brien et al., 2005) with default parameters. Tetraploid cottons were divided into A and D subgenomes. Then, we combined these results into one file for searching orthologous gene ID.

Based on gene expression data, networks of co-expressed genes of three cotton species (*G. arboreum*_ICR, *G. hirsutum_*TM-1_ICR, and *G. hirsutum*_ZM24_ICR) were constructed using Pearson’s correlation coefficient (PCC) values between pairs of genes and visualized by using JavaScript Cytoscape.js. For a given query gene, the network of top 20 target genes with the highest correlation values with the query gene is shown. In addition, a summary table of all co-expressed genes and corresponding functional annotations is provided below the network.

## Results and Discussion

### Overview of Website Structure and Function

**Figure 1:**
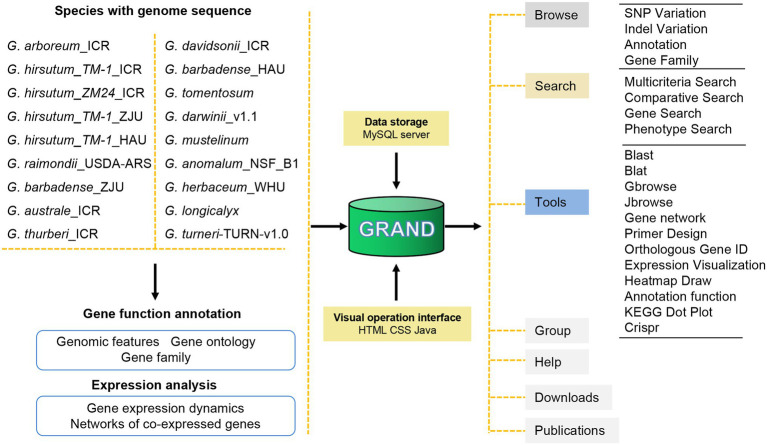
Schematic of GRAND database structure and web interface features. GRAND gathers 18 genomes, four transcriptomes of cotton and associated genome variations, and annotations data. All data are stored in a MySQL database.

To provide users with a wealth of information about cotton, the GRAND database was built containing the latest and most comprehensive *Gossypium* genomic/transcriptomic datasets (including 18 assembled genomes and four transcriptomes; Figure 1; Table 1). The main structure of GRAND is shown in Figure 1 with four major modules: Browse, Search, Tools, and Download. GRAND provides search functions for various genomic information, including gene annotation information (KOG, GO, KEGG, and NR), gene sequences, genome variations (SNPs and INDELs), and expressional profiles and gene families, by entering a chromosomal region or longest transcripts ID. GRAND also integrated the genome visualization tools Gbrowse (Stein et al., 2002) and Jbrowse (Buels et al., 2016), allowing users to instantly browse, visualize, and retrieve sequence data and offer gene co-expression networks for different developmental stages and tissues/organs. Moreover, GRAND provides a suite of the toolbox for online analysis, such as BLAST- and BLAT-based sequence comparisons, orthologous gene ID across different species and PCR primer design (Untergasser et al., 2012). Besides, users can download cotton data selectively or in full. Tutorials for using all the tools in the database are provided in the Help module. This information in GRAND will be useful for both dry lab and wet lab biologists.

### Browse Functions in GRAND

**Figure 2:**
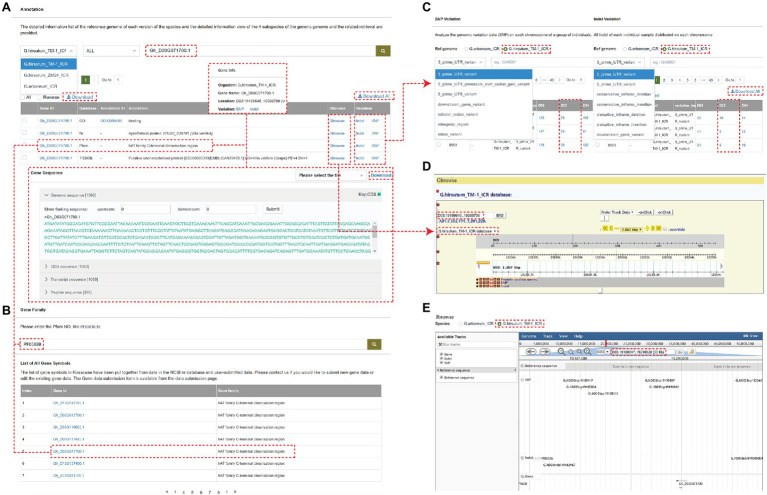
An example of a gene page showing multiple types of information associated with the gene. **(A)** The annotation information of gene “*Gh_D03G071700*.” **(B)** the gene “*Gh_D03G071700*” is annotated as the hAT family C-terminal dimerization region and can be also found at the end page by searching the gene family function panel with PF05699. **(C)** The SNP Variation or INDEL Variation analyzes the genomic variation data (SNP or INDEL) on each chromosome of a group of individuals. **(D,E)** The Gbrowse and Jbrowse page.

The browse detail page mainly includes the following modules: SNP Variation, INDEL Variation, Gene Annotation, and Gene Family. Users can search for the Nr, TrEMBL, KOG, KEGG, and GO annotations using Gene Annotation module. By cross-link, the genome variations related to each gene can be searched, including location, genome sequence, CDs sequence, transcript sequence, and peptide sequence (Figure 2A). Users can also quickly find related information about the gene family by searching for the target gene keywords (Figure 2B). The SNP or INDEL Variation shows the genome variations data (SNP or INDEL) on each chromosome of a group of individuals. Users can filter the data by reference genome, and SNP, or INDEL type (Figure 2C).

For example, the gene “*Gh_D03G071700*” is located on chromosome D03 of the *G. hirsutum_TM-1_ICR* genome and is annotated as the hAT family C-terminal dimerization region, which can be also found at the end page by searching the gene family module with PF05699. The SNP and Indel Variation can be directly queried by clicking on the Variation option and visualized by clicking on Gbrowse and Jbrowse (Figure 2), which are tools for displaying variations (SNPs and INDELs) and genes (structure) of the cotton individuals on chromosomes. The Gbrowse detail page includes the following basic information of this gene: name, position in the scaffold, length, CDS parts, and sequence. The detail page of Jbrowse is displayed in a popup window showing information about the gene, SNP, and INDEL in the 30 zkb region around this gene.

### Search Functions in GRAND

**Figure 3:**
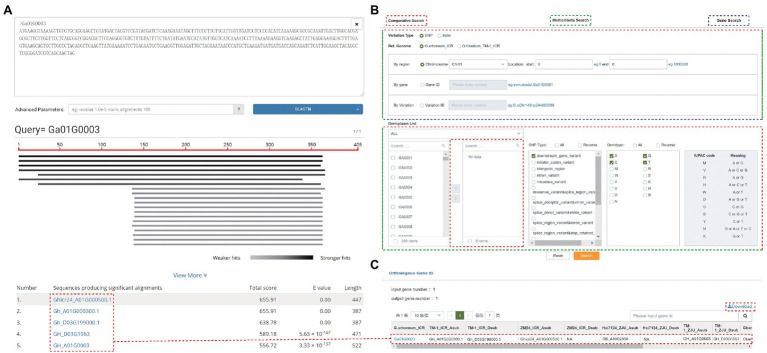
Search functions in GRAND. **(A)** The results of a BLAST search. **(B)** The Search page contains Comparative Search, Multicriteria Search, and Gene Search. **(C)** The orthologous gene ID function allows searching for orthologous gene IDs among different species and versions of cotton genome.

GRAND allows users to perform both BLAST and BLAT searches to rapidly align sequences to the genome. BLAST search implemented in GRAND using SequenceServer (Priyam et al., 2019) provides an interface with text-based and interactive visual outputs to search against nucleotide sequences and/or protein sequences, including BLASTn, BLASTp, BLASTx, tBLASTx, and tBLASTn programs. GRAND currently has a BLAST database for whole-genome sequences, CDSs, and predicted proteins for each reference genome assembly. Pasting the DNA/Protein sequences in the query box or uploading a fasta file is acceptable. The search result displayed on a result page comprises two parts: “Graphical view” and “List view” (Figure 3A). The Graphical view presents a brief graphical view of the BLAST results by the chart. The List view is a table showing detailed information on the alignment by the BLAST program, such as gene ID, total score, e-value, and length. The sequence of FASTA files can be annotated by comparing with database (Nr_vs_GO, KEGG, COG, SwissProt, TrEMBL, KOG and Pfam; Ashburner et al., 2000; Tatusov et al., 2000; Koonin et al., 2004; El-Gebali et al., 2019) in the “Anno function” section. The results of the KEGG annotation can be visualized in the “KEGG Dot Plot” section. Besides, to make it easier for users to quickly search for data of interest, the current version of GRAND has four submodules under the “Search” module. (i) Multicriteria Search. Search the genome variations (SNPs and INDELs) of each individual in this database using gene, region, or variation. Data can be filtered by variation type and genotype. (ii) Phenotype Search. The phenotype data of fiber-related traits, floral traits, seed-related traits, and other traits for cotton species. (iii) Comparative Search. Search and compare the genome variations (SNPs and INDELs) of two or more cotton individuals in this database using gene, region, and variation. (iv) Gene Search. Search and achieve gene annotation (KOG, GO, KEGG, and NR), gene structure, sequences by inputting the chromosomal region and gene ID. The SNPs, and INDELs can be also searched by cross-link (Figure 3B). After searching, a new webpage will pop out and display all the matched results. The details of each matched result can be viewed by clicking on it. The Orthologous Gene ID function can obtain orthologous gene IDs among different cotton species and different versions of cotton. For example, the orthologous genes ID of gene “*Ga01G0003*” are “*Gh_A01G000300*,” “*Gh_D03G199000*,” “*Ghicr24_A01G000500*,” and “*GB_A10G2858*” in other cotton species, respectively (Figure 3C). This result was consistent with the result of the BLAST above.

### Tools for Online Analysis in GRAND

**Figure 4:**
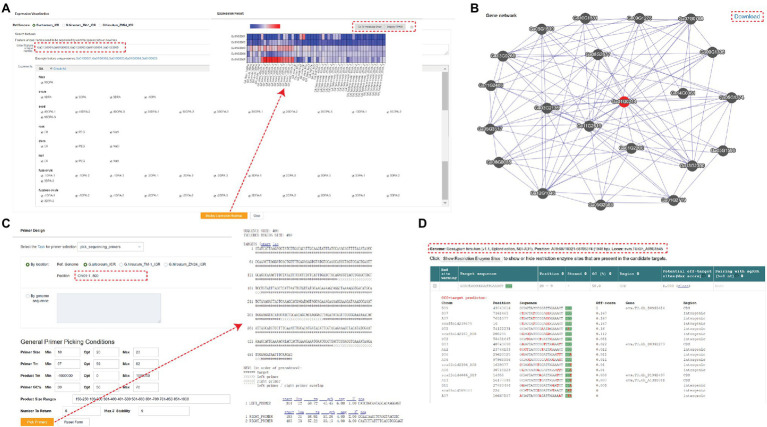
Tools in GRAND. **(A)** A heatmap showing gene expression search results. Each row represents the expression data of a gene across many samples, and each column shows the expression in a particular sample. **(B)** Co-expression network search results. The network shows the top 20 nodes near the target gene, and the corresponding information can be viewed by clicking. **(C)** Primer design page. **(D)** TargetDesign tool for designing primers for CRISPR/Cas9/Cpf1 genome editing.

In addition to the modules mentioned above, GRAND also offers several additional tools. The “Expression Visualization” section shows the expression profiles in different tissues, and users can perform the analysis by partial selection or by selecting all. The results are presented as heatmaps and the expression values (FPKM) for each data set are displayed in the table at the bottom (Figure 4A). Users can also import the results generated above into the “Heatmap Draw” section for further adjustment and embellishment. Gene network analysis can be used to identify related genes in the same biological processes or pathways. Networks of co-expressed genes are constructed based on inter-gene expression data using Pearson’s correlation coefficient (PCC) between genes (Langfelder and Horvath, 2008). Enter the gene ID and set the PCC value threshold to visualize the top 20 target genes with the highest correlation value with the query gene, and click on any co-expressed genes in the network to view their co-expression network. In addition, a summary table of all co-expressed genes and corresponding functional annotations is provided below the network. Links to basic information about the genes are created for each target gene in the summary table (Figure 4B). GRAND database provides primer design function based on gene sequences from cotton (Figure 4C). Users can also design primers for CRISPR/Cas9/Cpf1 genome editing using the targetDesign tool *via* the website link (Figure 4D; Xie et al., 2017). Additionally, we provide an FTP server to store all the publicly released datasets used in GRAND, with an enhanced user interface, text preview, and directory download.

### Download Functions in GRAND

The download page provides users with selective FTP download for genome sequences and their annotation information, transcriptomics data, CDS, protein, etc.

### Limitations

GRAND currently still has some limitations. For example, only cotton genomic, transcriptomic and phenotypic data were collected here, some additional data, such as cotton molecular markers and metabolic data need to be expanded in the future. Moreover, there is no sequence feature extraction tool in the current database.

## Conclusion and Perspectives

GRAND provides access to the various data, such as the genomic, transcriptomic, and phenotypic data for cotton. It can be browsed, mined, analyzed, and even downloaded. Moreover, GRAND provides an interface to visualize genomes, annotated genes, gene expression, and networks of co-expressed genes. The plenty available data contributes to highly resolving comparative genomics studies that shed light on the evolution and diversification of the various cotton species. During subsequent upgrades, the GRAND database will add sequence feature extraction tool and newly generated cotton data.

## Data Availability Statement

The original contributions presented in the study are included in the article/supplementary material, further inquiries can be directed to the corresponding author.

## Author Contributions

ZZ and LF wrote the initial draft. ZZ, ZhY, ZuY, MC and LF collected, curated, and formatted the data, and tested the GRAND and the use examples. All authors were involved in reviewing and editing the manuscript.

## Funding

This work was supported by funding from the National Natural Science Foundation of China (grant 31621005) and Xinjiang Changji Hui Autonomous Prefecture Science and Technology Projects (grant 2021Z01).

## Conflict of Interest

The authors declare that the research was conducted in the absence of any commercial or financial relationships that could be construed as a potential conflict of interest.

## Publisher’s Note

All claims expressed in this article are solely those of the authors and do not necessarily represent those of their affiliated organizations, or those of the publisher, the editors and the reviewers. Any product that may be evaluated in this article, or claim that may be made by its manufacturer, is not guaranteed or endorsed by the publisher.

## Footnotes

^1^ http://cotton.zju.edu.cn/

^2^ http://cole-trapnelllab.github.io/cufflinks/

## References

[ref1] Ashburner M. Ball C. A. Blake J. A. Botstein D. Butler H. Cherry J. M.. (2000). Gene ontology: tool for the unification of biology. The Gene Ontology Consortium. Nat. Genet. 25, 25–29. doi: 10.1038/75556, PMID: 10802651 PMC3037419

[ref2] Buels R. Yao E. Diesh C. M. Hayes R. D. Munoz-Torres M. Helt G.. (2016). JBrowse: a dynamic web platform for genome visualization and analysis. Genome Biol. 17:66. doi: 10.1186/s13059-016-0924-1, PMID: 27072794 PMC4830012

[ref3] Cai Y. Cai X. Wang Q. Wang P. Zhang Y. Cai C.. (2019). Genome sequencing of the Australian wild diploid species *Gossypium australe* highlights disease resistance and delayed gland morphogenesis. Plant Biotechnol. J. 18, 814–828. doi: 10.1111/pbi.13249, PMID: 31479566 PMC7004908

[ref4] Chen Z. J. Sreedasyam A. Ando A. Song Q. de Santiago L. M. Hulse-Kemp A. M.. (2020). Genomic diversifications of five *Gossypium* allopolyploid species and their impact on cotton improvement. Nat. Genet. 52, 525–533. doi: 10.1038/s41588-020-0614-5, PMID: 32313247 PMC7203012

[ref5] Du X. Huang G. He S. Yang Z. Sun G. Ma X.. (2018). Resequencing of 243 diploid cotton accessions based on an updated A genome identifies the genetic basis of key agronomic traits. Nat. Genet. 50, 796–802. doi: 10.1038/s41588-018-0116-x, PMID: 29736014

[ref6] El-Gebali S. Mistry J. Bateman A. Eddy S. R. Luciani A. Potter S. C.. (2019). The Pfam protein families database in 2019. Nucleic Acids Res. 47, D427–D432. doi: 10.1093/nar/gky995, PMID: 30357350 PMC6324024

[ref600] Grover C. E. Pan M. Yuan D. Arick M. A. Hu G. Brase L.. (2020). The *Gossypium longicalyx* genome as a resource for cotton breeding and evolution. G3-Genes, Genom. Genet. 10, 1457–1467. doi: 10.1534/g3.120.401050, PMID: 32122962 PMC7202014

[ref700] Grover C. E. Yuan D. Arick M. A. Miller E. R. Hu G. Peterson D. G.. (2021). The *Gossypium anomalum* genome as a resource for cotton improvement and evolutionary analysis of hybrid incompatibility. G3-Genes, Genom. Genet. 11. doi: 10.1093/g3journal/jkab319, PMID: 34549783 PMC8527517

[ref7] Hu Y. Chen J. Fang L. Zhang Z. Ma W. Niu Y.. (2019). *Gossypium barbadense* and *Gossypium hirsutum* genomes provide insights into the origin and evolution of allotetraploid cotton. Nat. Genet. 51, 739–748. doi: 10.1038/s41588-019-0371-5, PMID: 30886425

[ref8] Huang G. Wu Z. Percy R. G. Bai M. Li Y. Frelichowski J. E.. (2020). Genome sequence of *Gossypium herbaceum* and genome updates of *Gossypium arboreum* and *Gossypium hirsutum* provide insights into cotton A-genome evolution. Nat. Genet. 52, 516–524. doi: 10.1038/s41588-020-0607-4, PMID: 32284579 PMC7203013

[ref9] Kim D. Pertea G. Trapnell C. Pimentel H. Kelley R. Salzberg S. L. (2013). TopHat2: accurate alignment of transcriptomes in the presence of insertions, deletions and gene fusions. Genome Biol. 14:R36. doi: 10.1186/gb-2013-14-4-r36, PMID: 23618408 PMC4053844

[ref10] Koonin E. V. Fedorova N. D. Jackson J. D. Jacobs A. R. Krylov D. M. Makarova K. S.. (2004). A comprehensive evolutionary classification of proteins encoded in complete eukaryotic genomes. Genome Biol. 5:R7. doi: 10.1186/gb-2004-5-2-r7, PMID: 14759257 PMC395751

[ref11] Langfelder P. Horvath S. (2008). WGCNA: an R package for weighted correlation network analysis. BMC Bioinf. 9:559. doi: 10.1186/1471-2105-9-559, PMID: 19114008 PMC2631488

[ref12] O’Brien K. P. Remm M. Sonnhammer E. L. (2005). Inparanoid: a comprehensive database of eukaryotic orthologs. Nucleic Acids Res. 33, D476–D480. doi: 10.1093/nar/gki107, PMID: 15608241 PMC540061

[ref13] Peng Z. Li H. Sun G. Dai P. Geng X. Wang X.. (2021). CottonGVD: a comprehensive genomic variation database for cultivated cottons. Front. Plant Sci. doi: 10.3389/fpls.2021.803736 PMC8724205 34992626

[ref14] Priyam A. Woodcroft B. J. Rai V. Moghul I. Munagala A. Ter F.. (2019). Sequenceserver: a modern graphical user interface for custom BLAST databases. Mol. Biol. Evol. 36, 2922–2924. doi: 10.1093/molbev/msz185, PMID: 31411700 PMC6878946

[ref15] Shen C. Wang N. Zhu D. Wang P. Wang M. Wen T.. (2021). *Gossypium tomentosum* genome and interspecific ultra-dense genetic maps reveal genomic structures, recombination landscape and flowering depression in cotton. Genomics 113, 1999–2009. doi: 10.1016/j.ygeno.2021.04.036, PMID: 33915244

[ref16] Stein L. D. Mungall C. Shu S. Q. Caudy M. Mangone M. Day A.. (2002). The generic genome browser: a building block for a model organism system database. Genome Res. 12, 1599–1610. doi: 10.1101/gr.403602, PMID: 12368253 PMC187535

[ref17] Tatusov R. L. Galperin M. Y. Natale D. A. Koonin E. V. (2000). The COG database: a tool for genome-scale analysis of protein functions and evolution. Nucleic Acids Res. 28, 33–36. doi: 10.1093/nar/28.1.33, PMID: 10592175 PMC102395

[ref18] Udall J. A. Long E. Hanson C. Yuan D. Ramaraj T. Conover J. L.. (2019). *De novo* genome sequence assemblies of *Gossypium raimondii* and *Gossypium turneri*. G3 9, 3079–3085. doi: 10.1534/g3.119.400392, PMID: 31462444 PMC6778788

[ref19] Untergasser A. Cutcutache I. Koressaar T. Ye J. Faircloth B. C. Remm M.. (2012). Primer3-new capabilities and interfaces. Nucleic Acids Res. 40:e115. doi: 10.1093/nar/gks596, PMID: 22730293 PMC3424584

[ref20] Wang D. Fan W. Guo X. Wu K. Zhou S. Chen Z.. (2020). MaGenDB: a functional genomics hub for Malvaceae plants. Nucleic Acids Res. 48, D1076–D1084. doi: 10.1093/nar/gkz953, PMID: 31665439 PMC7145696

[ref21] Wang M. Tu L. Yuan D. Zhu D. Shen C. Li J.. (2019). Reference genome sequences of two cultivated allotetraploid cottons, *Gossypium hirsutum* and *Gossypium barbadense*. Nat. Genet. 51, 224–229. doi: 10.1038/s41588-018-0282-x, PMID: 30510239

[ref22] Xie X. Ma X. Zhu Q. Zeng D. Li G. Liu Y. G. (2017). CRISPR-GE: a convenient software toolkit for CRISPR-based genome editing. Mol. Plant 10, 1246–1249. doi: 10.1016/j.molp.2017.06.004, PMID: 28624544

[ref23] Yang Z. Ge X. Li W. Jin Y. Liu L. Hu W.. (2021). Cotton D genome assemblies built with long-read data unveil mechanisms of centromere evolution and stress tolerance divergence. BMC Biol. 19:115. doi: 10.1186/s12915-021-01041-0, PMID: 34082735 PMC8176745

[ref24] Yang Z. Ge X. Yang Z. Qin W. Sun G. Wang Z.. (2019). Extensive intraspecific gene order and gene structural variations in upland cotton cultivars. Nat. Commun. 10:2989. doi: 10.1038/s41467-019-10820-x, PMID: 31278252 PMC6611876

[ref25] You Q. Xu W. Zhang K. Zhang L. Yi X. Yao D.. (2017). ccNET: database of co-expression networks with functional modules for diploid and polyploid *Gossypium*. Nucleic Acids Res. 45, D1090–D1099. doi: 10.1093/nar/gkw910, PMID: 28053168 PMC5210623

[ref26] Yu J. Jung S. Cheng C. H. Ficklin S. P. Lee T. Zheng P.. (2014). CottonGen: a genomics, genetics and breeding database for cotton research. Nucleic Acids Res. 42, D1229–D1236. doi: 10.1093/nar/gkt1064, PMID: 24203703 PMC3964939

[ref27] Zhu T. Liang C. Meng Z. Sun G. Meng Z. Guo S.. (2017). CottonFGD: an integrated functional genomics database for cotton. BMC Plant Biol. 17:101. doi: 10.1186/s12870-017-1039-x, PMID: 28595571 PMC5465443

